# Discordance between immunofixation and free light chain assays in multiple myeloma: a retrospective analysis and evaluation of the heavy/light chain assay for disease monitoring

**DOI:** 10.1007/s44313-026-00130-9

**Published:** 2026-03-06

**Authors:** Oytip Nathalang, Yupapin Onthong, Chonlada Laoruangroj, Dollapak Apipongrat

**Affiliations:** 1 Graduate Program in Medical Technology, Faculty of Allied Health Sciences, Thammasat University, Pathumtani, Thailand; 2 Division of Hematology, Department of Medicine, Phramongkutklao Hospital, Bangkok, Thailand; 3 Department of Pathology, Phramongkutklao College of Medicine, Ratchathewi District, 316 Ratchawithi Road, Bangkok, 10400 Thailand

**Keywords:** Multiple myeloma, Monoclonal protein, Heavy/light chain assay, Immunofixation electrophoresis, Serum-free light chain

## Abstract

**Background:**

Accurate detection and monitoring of monoclonal proteins in multiple myeloma (MM) are critical for diagnosis and treatment guidance. Conventional assays such as serum protein electrophoresis (SPEP), immunofixation electrophoresis (SIFE), and serum-free light chain (SFLC) testing can yield discordant or inconclusive results, particularly in patients with low M-protein levels.

**Objectives:**

To evaluate the concordance and discordance between SIFE and SFLC assays in MM and to assess the clinical utility of the heavy/light chain (HLC) assay as a complementary quantitative tool.

**Methods:**

We retrospectively analyzed 1,310 MM patient samples. The concordance and discordance between SIFE and SFLC were assessed, and the analytical performance of HLC measurements was evaluated relative to conventional assays.

**Results:**

The overall discordance rate between SIFE and SFLC was 26.9%, with SIFE–/SFLC + most frequent in light chain MM and SIFE + /SFLC– observed in patients who achieved a very good partial response (VGPR). HLC strongly correlated with total immunoglobulins (combined immunoglobulin G [IgG]/immunoglobulin A [IgA], *R*^*2*^ = 0.99) and SPEP (*R*^*2*^ = 0.94). HLC ratios demonstrated excellent concordance with SIFE (*κ* = 0.83, 95% confidence interval [CI] 0.72–0.93) but poor agreement with SFLC ratios (*κ* = 0.01, 95% CI − 0.18–0.20). Using the HLC response model, 15 patients classified as having VGPR by the International Myeloma Working Group (IMWG) criteria were reclassified as having a partial response due to a dHLC reduction of < 90%.

**Conclusions:**

This large retrospective study provides a comprehensive evaluation of SIFE and SFLC discordance in MM, offering real-world evidence of their complementary diagnostic and monitoring roles. These findings support the integration of HLC testing into existing response assessment strategies to enhance the accuracy of treatment evaluation and the interpretation of discordant results.

**Supplementary Information:**

The online version contains supplementary material available at 10.1007/s44313-026-00130-9.

## Introduction

Multiple myeloma (MM) is a clonal plasma cell disorder characterized by the production of monoclonal immunoglobulins (M-proteins), which can be detected and monitored using various laboratory assays [1–3]. Accurate assessment of M proteins and their related components plays an important role in disease diagnosis, evaluation of treatment response, and monitoring of disease relapse [1–4]. Conventional techniques such as serum protein electrophoresis (SPEP) and serum immunofixation electrophoresis (SIFE) have long served as cornerstone methods for quantifying and identifying M proteins [5]. Recently, turbidimetric immunoassays for detecting and quantifying serum-free light chains (SFLC) have become widely available. Measurement of free kappa (κ) and lambda (λ) light chains provides complementary information to SPEP and SIFE, particularly in light chain–only myeloma and nonsecretory disease [6–8]. The SFLC assay has proven valuable in diagnosis, disease monitoring, and prognosis, offering higher sensitivity than conventional electrophoretic techniques for detecting minimal residual disease (MRD) or early relapse compared with conventional electrophoretic techniques [6–10].

Despite their utility, discrepancies between SIFE and SFLC results are frequently encountered in clinical practice [11–15]. Such discordant findings may result from biological variations in M-protein secretion, differences in analytical sensitivity, or interference from polyclonal background immunoglobulins [9, 11, 12]. These discordances can pose significant challenges in clinical management, potentially leading to underestimation of disease burden, misclassification of treatment response, or delayed detection of relapse. Therefore, understanding and addressing these inconsistencies are essential for accurate disease monitoring in MM.

The heavy/light chain (HLC) immunoassay (Hevylite™ assay, The Binding Site Group Ltd., Birmingham, UK) is a novel method for quantifying specific immunoglobulin isotypes based on their heavy- and light-chain pairings (e.g., immunoglobulin G kappa [IgGκ]/immunoglobulin G lambda [IgGλ], immunoglobulin A kappa [IgAκ]/immunoglobulin A lambda [IgAλ], immunoglobulin M kappa [IgMκ]/immunoglobulin M lambda [IgMλ]) [16–18]. By providing both quantitative measurements and κ/λ ratios within each immunoglobulin class, the HLC assay offers additional insights into clonality and disease burden, potentially enabling more precise detection of early disease relapse and MRD [16, 18–24]. Several studies have suggested that HLC measurement may complement conventional assays by enhancing disease monitoring, particularly in immunoglobulin A (IgA) myeloma, in which M-proteins often migrate to the β-region and may be difficult to quantify accurately using electrophoretic methods [25–27]. These findings suggest that HLC assays provide a more comprehensive assessment of MM and may benefit patients with discordant conventional laboratory results, supporting their use as complementary tools in disease monitoring.

In this study, we aimed to explore the characteristics of patients with MM presenting with varying laboratory findings, with a focus on cases showing discordant results between SIFE and SFLC assays, using a large retrospective real-world dataset. Clinical data, including response status over a three-year follow-up period, were comprehensively evaluated in conjunction with laboratory findings to investigate the relationship between SIFE/SFLC discordance and treatment response status in routine clinical practice. Although several studies have described the analytical characteristics and clinical value of the HLC assay [16–24], data on its clinical interpretability in routine practice and its impact on response assessment remain limited. Therefore, the clinical performance of the HLC assay was further evaluated, and its correlation and agreement with conventional methods were systematically assessed to determine its potential role as a complementary tool for routine MM monitoring.

## Materials and methods

### Study population

We retrospectively reviewed the medical records of patients whose serum samples were submitted for SPEP, SIFE, and SFLC testing at the Laboratory of Special Hematology, Division of Hematology, Department of Medicine, Phramongkutklao Hospital, Thailand, between January 2022 and December 2024. Patients without MM were excluded from this study. Demographic and clinical characteristics, including age at diagnosis, sex, MM isotype, disease stage, treatment regimen, and response status, were recorded. SPEP and corresponding M-protein quantification were performed by agarose gel electrophoresis using an SPIFE® Split Beta SPE kit on a SPIFE 3000 system (Helena Laboratories, Beaumont, TX, USA). SPIFE was performed using the SPIFE® ImmunoFix-6 kit (Helena Laboratories, Beaumont, TX, USA), which utilizes monospecific antisera against IgG, IgM, and IgA heavy chains, as well as κ and λ light chains, on the same analyzer. SFLC κ and λ levels were measured by turbidimetry using the Optilite® Freelite™ Kappa Free and Freelite™ Lambda Free kits on an Optilite® Analyzer (The Binding Site Group Ltd., Birmingham, UK).

To evaluate the clinical performance of the HLC assay, 146 leftover serum samples from 38 patients with intact immunoglobulin MM were analyzed. These consisted of 38 samples collected at diagnosis and 108 samples obtained during treatment from patients who had achieved at least a very good partial response (VGPR) according to the International Myeloma Working Group (IMWG) uniform response criteria [28]. All serum samples were aliquoted and stored at − 20 °C until analysis.

### Concordance and discordance between SIFE and SFLC

Monoclonal gammopathy was defined as the presence of a restricted monoclonal band in the gamma region or prominent bands in other protein regions of the serum as detected by SPEP and confirmed by SIFE. SFLC results were considered negative if the κ/λ ratio was within the normal range (0.26–1.65), whereas ratios < 0.26 or > 1.65 were considered abnormal. Concordance between SIFE and SFLC was defined as similar results on both assays (SIFE +/SFLC + or SIFE −/SFLC −), whereas discordance was defined as either a positive SIFE with a normal SFLC ratio (SIFE +/SFLC −) or a negative SIFE with an abnormal SFLC ratio (SIFE −/SFLC +).

### Measurement of heavy/light chain

Immunoglobulin HLC pairs, including IgG κ, IgGλ, IgAκ, and IgAλ, were measured using a latex-enhanced immunoassay with the Hevylite™ assay on the Optilite® Analyzer (The Binding Site Group Ltd., Birmingham, UK). The HLC κ/λ ratio (HLCr) and the difference between involved HLC (iHLC) and uninvolved HLC (uHLC), designated as dHLC, were subsequently calculated for each sample. According to a previous study by Koulieris et al. [29], the reference ranges were as follows: 0.40–0.98 g/dL for IgGκ; 0.20–0.57 g/dL for IgGλ; 0.98–2.75 for IgGκ/IgGλ HLCr; 0.05–0.28 g/dL for IgAκ; 0.04–0.20 g/dL for IgAλ; and 0.80–2.04 for IgAκ/IgAλ HLCr. Values outside these reference ranges were considered indicative of clonal proliferation.

For treatment response assessment using the HLC assay, we applied criteria adapted from previous studies [20]. Progressive disease (PD) was defined as a > 25% increase in dHLC, whereas stable disease (SD) was defined as a < 50% reduction in dHLC. Partial response (PR) corresponded to a 50–89% reduction in dHLC, and VGPR was defined as a ≥ 90% reduction in dHLC. Patients were classified as having achieved complete response (CR) or better if they had < 5% plasma cells on bone marrow examination, no evidence of plasmacytomas, and no detectable M-protein on SIFE or normalized HLCr.

### Statistical analysis

Statistical analyses were performed using SPSS version 21 (IBM Corp., Armonk, NY, USA) and GraphPad Prism version 9.0 (GraphPad Software, San Diego, CA, USA). Continuous variables are expressed as mean ± standard deviation (SD) or median and interquartile range (IQR), depending on the data distribution. Differences between two categorical groups were compared using the chi-square (*χ*^2^) test or Fisher’s exact test, as appropriate. Linear regression analysis was used to evaluate the linear relationship between test parameters and reported as *R*^*2*^*.* Correlation between test results was assessed using Spearman’s rank correlation coefficient (*r*ₛ) with a 95% confidence interval (CI). The average differences between results from different test parameters, along with the 95% CI of the limits of agreement (LOA), were calculated using Bland–Altman analysis [30]. Cohen’s kappa coefficient (*κ*) was applied to evaluate agreement between the two test parameters. A *P*-value of < 0.05 was considered statistically significant.

## Results

### Characteristics of MM patients across different laboratory findings

**Fig. 1:**
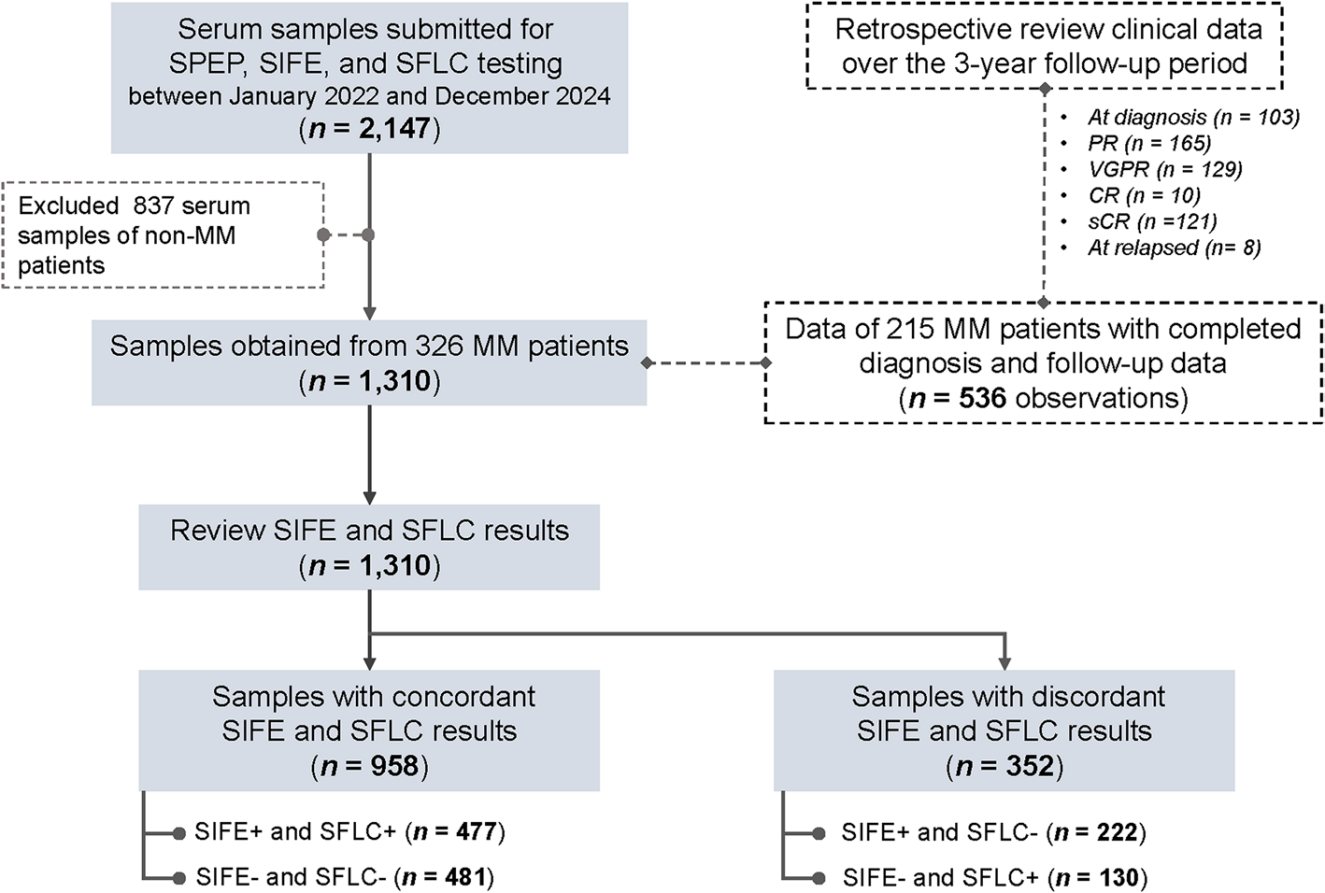
Schematic flowchart of sample selection and study design

A total of 2,147 serum samples submitted for SPEP, SIFE, and SFLC testing between January 2022 and December 2024 were reviewed. After excluding 837 non-myeloma samples, 1,310 samples from 326 patients with MM were analyzed. A schematic representation of this study is shown in Fig. 1.

The characteristics of the 1,310 serum samples are summarized in Table 1. Among these, 114 samples (8.7%) were from newly diagnosed (ND) patients and 1,196 (97.3%) were from follow-up cases. Concordant results between SIFE and SFLC were observed in 958 samples (71.3%), whereas 352 samples (26.9%) showed discordant results. Within the discordant group, 222 samples (17.0%) were SIFE +/SFLC– and 130 samples (9.9%) were SIFE–/SFLC +. The proportion of discordant samples was significantly higher in the follow-up cases than in the ND cases (*χ*^2^ = 9.08, df = 1, *P* = 0.002). Among patients with IgG-MM, discordance was significantly higher in IgGλ samples (*χ*^2^ = 22.57, df = 1, *P* = 0.001), whereas in IgA-MM, discordance was higher in IgAκ samples (*χ*^2^ = 5.96, df = 1, *P* = 0.015). As expected, among patients with light-chain MM, all discordant cases showed SIFE–/SFLC + results.

**Table 1:** Characteristics of 1,310 samples with concordant and discordant SIFE and SFLC ratio results

Classification	Number of samples	Samples with concordant results	Samples with discordant results, *n* (%)			χ^2^ (df)	*P*-value
			**SIFE + ** **/SFLC-**	**SIFE-** **/SFLC + **	**Total**		
**All samples, *n* (%)**	**1310**	**958 (73.1)**	**222 (17.0)**	**130 (9.9)**	**352 (26.9)**		
**Sample type, *n* (%)**							
Newly diagnosed cases	114	97 (85.1)	5 (4.4)	12 (10.5)	17 (14.9)	9.07 (1)	*0.002*
Follow-up cases	1196	861 (72.0)	217 (18.1)	118 (9.9)	335 (28.0)		
**Clonal isotype, *n* (%)**							
IgG Kappa	475	376 (79.2)	86 (18.1)	13 (2.7)	99 (20.8)	22.57 (1)	< *0.001*
IgG Lambda	276	173 (62.7)	94 (34.1)	9 (3.3)	103 (37.3)		
IgA Kappa	155	120 (77.4)	20 (12.9)	15 (9.7)	35 (22.6)	5.96 (1)	*0.015*
IgA Lambda	157	138 (86.7)	18 (11.5)	1 (0.6)	19 (12.1)		
IgM Kappa	5	5 (100.0)	0 (0.0)	0 (0.0)	0 (0.0)	2.85 (1)	0.091*
IgM Lambda	4	2 (50.0)	2 (50.0)	0 (0.0)	2 (50.0)		
IgD Kappa	0	0 (0.0)	0 (0.0)	0 (0.0)	0 (0.0)	N/A	N/A
IgD Lambda	15	13 (86.7)	2 (13.3)	0 (0.0)	2 (13.3)		
Kappa light chains	157	90 (57.3)	0 (0.0)	67 (42.7)	67 (42.7)	N/A	N/A
Lambda light chains	66	41 (62.1)	0 (0.0)	25 (37.9)	25 (37.9)	N/A	N/A
**Disease status, *n* (%)**							
At diagnosis	114	97 (85.1)	5 (4.4)	12 (10.5)	17 (14.9)		
SD	25	24 (96.0)	0 (0.0)	1 (4.0)	1 (4.0)		
PD	43	22 (51.2)	13 (30.2)	8 (18.6)	21 (48.8)		
PR	337	281 (83.4)	27 (8.0)	29 (8.6)	56 (16.6)	N/A	N/A
VGPR	240	21 (8.8)	172 (71.7)	47 (19.5)	219 (91.2)		
CR or sCR	507	481 (94.9)	0 (0.0)	26 (5.1)	26 (5.1)		
Relapsed disease	44	32 (72.7)	5 (11.4)	7 (15.9)	12 (27.3)		

CR, complete remission; Ig, immunoglobulin; N/A, not applicable; PD, progressive disease; PR, partial response; sCR, stringent complete remission; SD, stable disease; SFLC, serum free light chain; SIFE, serum immunofixation electrophoresis; VGPR, very good partial response.

### Distribution of concordant and discordant SIFE and SFLC results during follow-up

In this analysis, 215 patients with MM with complete diagnostic and follow-up data (536 observations) were retrospectively reviewed to evaluate clinical characteristics and treatment responses across disease stages. Demographic and clinical characteristics of the cohort are summarized in Supplementary Table S1. In this cohort, 120 (55.8%) patients were male and 95 (44.2%) were female. The mean age (± SD) was 62.4 ± 12.2 years, and 125 patients (58.1%) were eligible for autologous stem cell transplantation (ASCT; age < 65 years). Most patients (53.6%) had MM classified as International Staging System (ISS) stage III [31], and 69.3% received a bortezomib-based treatment regimen.

**Fig. 2:**
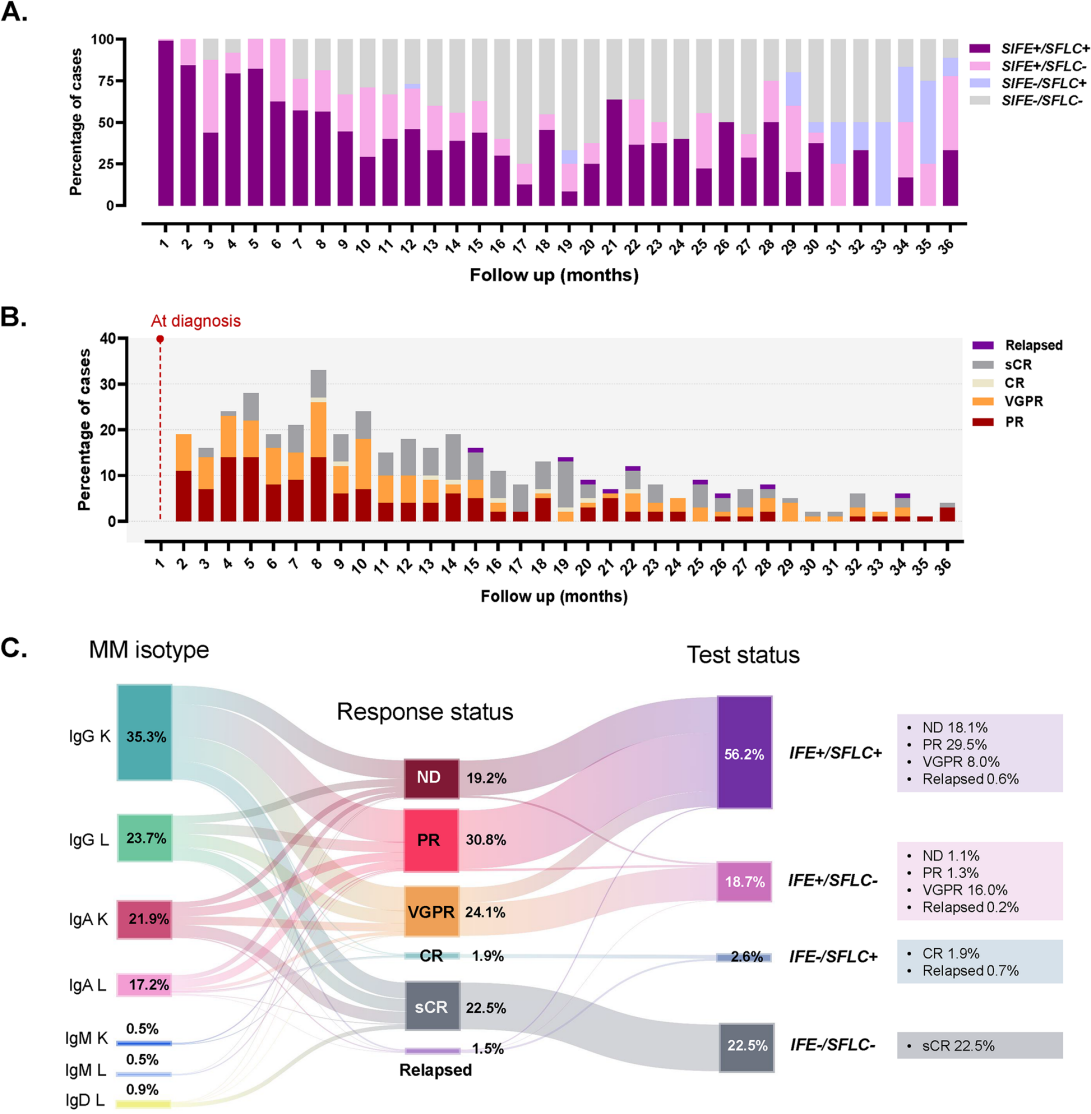
Distribution of concordant and discordant SIFE/SFLC results and treatment response during follow-up in 215 patients with multiple myeloma (MM). (A) Temporal distribution of SIFE/SFLC concordant (SIFE +/SFLC +, SIFE −/SFLC −) and discordant (SIFE +/SFLC −, SIFE −/SFLC +) results across a 3-year follow-up period. (B) Distribution of treatment response status, including partial response (PR), very good partial response (VGPR), complete response (CR), stringent complete response (sCR), and relapse, aligned according to follow-up time points. (C) Sankey diagram illustrating the relationships among MM isotypes, treatment response status, and test concordance categories

When the samples were aligned over the 3-year follow-up period, the distributions of laboratory test results and treatment response statuses were analyzed, as summarized in Supplementary Table S2 and Fig. 2(A–C). Among all 536 samples analyzed, concordant SIFE +/SFLC + results accounted for 56.2%, whereas SIFE +/SFLC − and SIFE −/SFLC + discordant results were observed in 18.7% and 2.6% of samples, respectively. Double-negative (SIFE −/SFLC −) results comprised 22.5% of all samples. At the time of diagnosis, most samples (94.2%) showed concordant positivity (SIFE +/SFLC +). During follow-up, the proportion of SIFE +/SFLC − samples increased markedly, particularly in patients achieving a VGPR, where 86 of 129 samples (66.7%) showed this discordant pattern. In contrast, SIFE −/SFLC + results were infrequent (2.6% of all samples) and were mainly observed in the CR (100.0%) and relapse (50.0%) groups, as shown in Supplementary Table S2 and Fig. 2C**.** As expected, SIFE −/SFLC − results were predominant in the stringent CR (sCR) group (100.0%), reflecting effective disease suppression.

### Evaluation of the HLC assay in follow-up MM patients and its correlation with conventional tests

Among 38 patients with intact immunoglobulin MM enrolled in this cohort, the mean (± SD) age was 59.3 ± 12.4 years, and 34.2% were male. The most common clonal isotype was IgGκ (39.5%), followed by IgGλ (28.9%), IgAκ (21.1%), and IgAλ (10.5%). According to the ISS, 55.2% of patients were classified as stage III, 29.0% as stage II, and 15.8% as stage I. Most patients (73.7%) were eligible for ASCT, and 78.9% received a bortezomib-based first-line treatment regimen. The median follow-up duration was 11 months (range: 6–22 months; Supplementary Table S1).

**Fig. 3:**
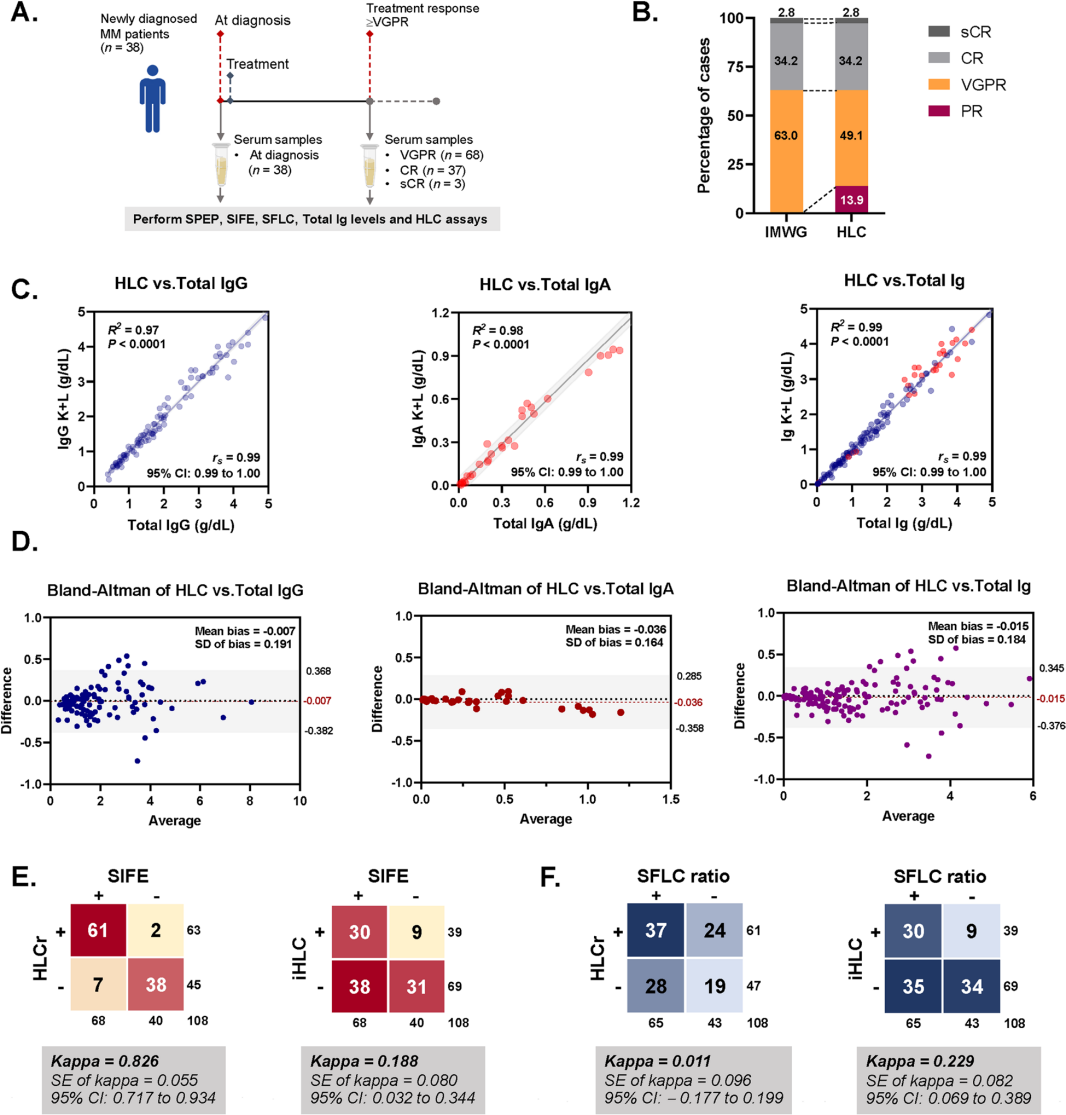
Evaluation of the heavy/light chain assay in patients with MM. **A** Study workflow showing sample collection from 38 patients with intact immunoglobulin MM, including samples at diagnosis (*n* = 38) and during treatment (≥ VGPR, *n* = 108). **B** Comparison of treatment response classification between IMWG criteria and HLC-based assessment. **C** Correlation plots showing strong linear relationships between HLC and total immunoglobulin levels for IgG, IgA, and overall immunoglobulins (*R*^*2*^ = 0.97, 0.98, and 0.99, respectively). The gray-shaded area represents the 95% CI. **D** Bland–Altman analyses demonstrating good agreement between HLC and total immunoglobulin measurements. The gray-shaded area represents the 95% LOA. **E** Agreement between HLC parameters (HLCr and iHLC) and SIFE results. **F** Agreement between HLC parameters (HLCr and iHLC) and SFLC ratio results

A total of 146 serum samples from 38 patients were analyzed using the HLC assay. Among these, 38 samples were obtained at diagnosis and 108 were obtained during treatment from patients who achieved at least VGPR (≥ VGPR, Fig. 3A). The treatment responses determined by the HLC assay closely paralleled those defined by the IMWG criteria. Based on the IMWG classification, 63.0%, 34.2%, and 2.8% of patients achieved VGPR, CR, and sCR, respectively. When evaluated using the HLC assay, 49.1% achieved VGPR, 34.2% achieved CR, 2.8% achieved sCR, and 13.9% (15 of 108) achieved PR (Fig. 3B).

Strong positive correlations were observed between HLC and total immunoglobulin concentrations for both IgG (*R*^*2*^ = 0.97, *r*_*s*_ = 0.99, 95% CI: 0.99–1.00) and IgA (*R*^*2*^ = 0.98*, r*_*s*_ = 0.99, 95% CI: 0.99–1.00), as well as when all samples were combined (*R*^*2*^ = 0.99,* r*_*s*_ = 0.99, 95% CI: 0.99–1.00, Fig. 3C). Bland–Altman analysis further demonstrated good agreement between HLC and total immunoglobulin measurements, with minimal mean bias and a narrow LOA (Fig. 3D).

In agreement analysis, the HLCr showed excellent concordance with SIFE results (*κ* = 0.83, 95% CI 0.72–0.93) but poor agreement with the SFLC ratio (*κ* = 0.01, 95% CI − 0.18–0.20, Fig. 3E and F). These findings indicate that the HLC assay provides reliable quantification of immunoglobulin isotypes and demonstrates a strong correlation with electrophoretic results; however, its agreement with SFLC measurements remains limited.

**Fig. 4:**
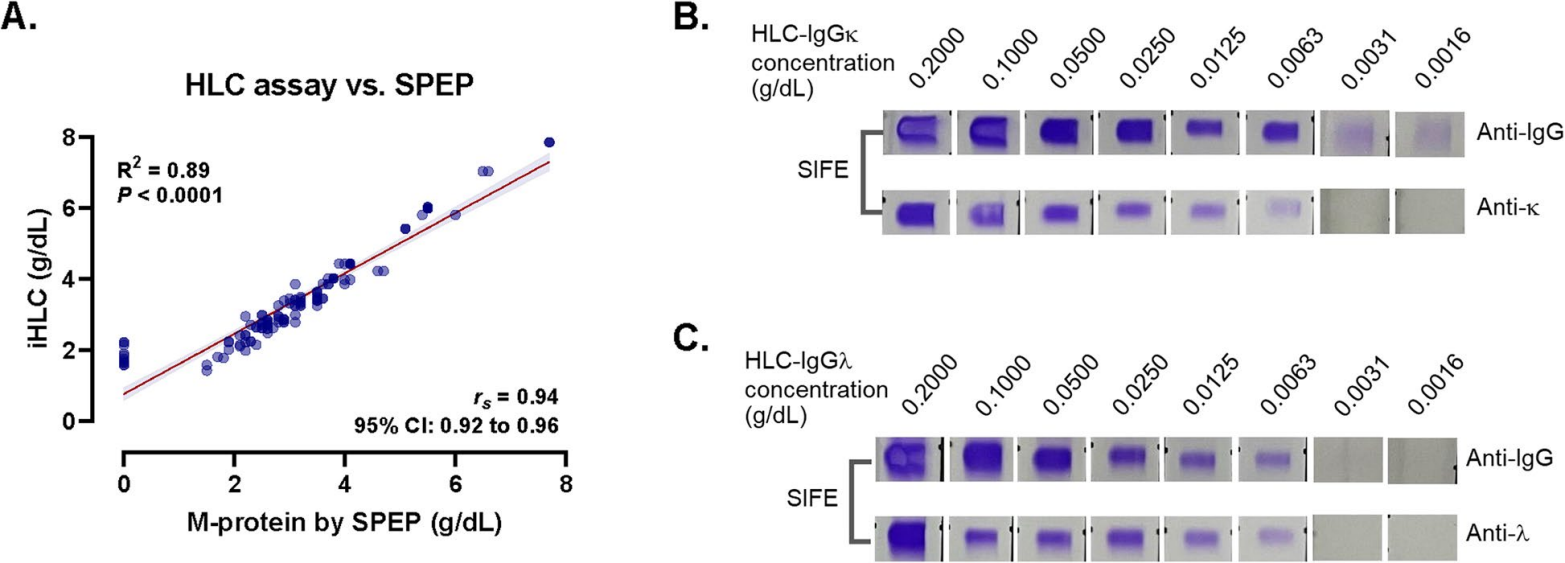
Comparison of M-protein quantification between the HLC assay and SPEP, and analytical sensitivity of the HLC assay versus SIFE in serially diluted IgGκ and IgGλ samples. (A) Correlation between M-protein concentrations measured by the HLC assay and SPEP in MM samples. (B–C) Analytical sensitivity comparison of SIFE and the HLC assay in serially diluted monoclonal (B) IgGκ and (C) IgGλ samples, starting from an initial concentration of 0.200 g/dL

Additionally, the linear correlation between M-protein quantification by SPEP and the HLC assay was evaluated. A strong positive correlation was observed between the two methods (*R*^*2*^ = 0.89, *r*ₛ = 0.94; 95% CI, 0.92–0.96). Notably, in several cases where the M-protein was undetectable by SPEP, it remained detectable by HLC (Fig. 4A).

### Detection limits between the HLC and SIFE assays

To evaluate the analytical sensitivity of the HLC and SIFE assays in detecting low concentrations of monoclonal immunoglobulins, two representative samples—one with IgGκ and another with IgGλ—were analyzed. The HLC concentrations were first determined, and the serum samples were subsequently diluted to an initial concentration of 0.200 g/dL, followed by serial two-fold dilutions to obtain final concentrations of 0.1000, 0.0500, 0.0250, 0.0125, 0.0063, 0.0031, and 0.0016 g/dL. SIFE was then performed, and monoclonal bands remained visually detectable at concentrations below 0.0063 g/dL for both IgGκ and IgGλ samples (Figs. 4B and C). In contrast, the HLC assay yielded quantifiable results across all tested dilutions, consistent with its lower limits of detection—0.0009 g/dL for IgGκ and 0.0005 g/dL for IgGλ [17]—thereby demonstrating superior analytical sensitivity compared with SIFE.

## Discussion

Accurate detection and monitoring of monoclonal proteins in MM are essential for diagnosis, treatment guidance, and response assessment. Conventional laboratory tests, including SPEP, SIFE, and SFLC, can yield discordant or inconclusive results, particularly in patients with low M-protein levels, posing challenges for clinical interpretation in routine practice. In this large, real-world, retrospective cohort study with longitudinal clinical follow-up, we systematically evaluated patterns of concordance and discordance between SIFE and SFLC across different treatment response categories. Furthermore, we assessed the clinical performance of the HLC assay and its correlation and agreement with conventional methods to determine its potential role as a complementary tool for MM monitoring.

Discordant rates between SIFE and SFLC results have been reported to range from 17 to 50% in previous studies [11–15]. In the present study, the discordance rate was 26.9%, which is comparable to the 27.0% reported by Singh G [13] and the 28.7% recently reported by Shastri et al. [11]. The proportion of discordant results was significantly higher in follow-up samples than in ND samples, reflecting dynamic changes in M-protein composition during treatment. As expected, all discordant results observed in light-chain MM were SIFE–/SFLC +, highlighting the superior analytical sensitivity of the SFLC assay for detecting low-level monoclonal light chains, particularly in post-treatment samples [5, 10, 32]. However, in intact immunoglobulin MM, discordance patterns varied by isotype, with a higher observed frequency of discordance in IgGλ and IgAκ subtypes. These findings suggest that biological and analytical variability between κ and λ isotypes, as well as the effects of polyclonal background suppression, may influence assay performance and interpretation.

When the cohort of patients with intact immunoglobulin MM was analyzed longitudinally, the distribution of concordant and discordant test results closely corresponded to the treatment response categories. Most SIFE +/SFLC– samples were derived from patients who achieved VGPR, indicating that SIFE may still detect residual monoclonal bands even after normalization of the SFLC ratio. Because intact immunoglobulins have a relatively long serum half-life (approximately 21 days for IgG and 5–6 days for IgA), whereas free light chains are cleared much more rapidly (half-life 2–6 h) [5], SFLC normalization typically occurs earlier following effective treatment. This kinetic difference may explain the temporary discordance observed between SIFE and SFLC results during treatment. Conversely, a subset of patients exhibited SIFE–/SFLC + results, reflecting the superior analytical sensitivity of the SFLC assay, which can detect trace levels of circulating monoclonal free light chains below the SIFE detection threshold [5, 8, 10, 32–34]. Such discordance is frequently observed in patients with light-chain MM, early relapse, or MRD, in whom the amount of monoclonal protein is insufficient to generate a visible band on electrophoresis [5, 8, 10, 32–34].

Evaluation of treatment response using HLC assay-based criteria demonstrated strong concordance with the conventional electrophoretic criteria defined by the IMWG. Most patients classified as having VGPR or CR by SIFE and clinical assessment were similarly categorized using HLC response criteria, supporting the reliability of the assay for quantitative assessment of monoclonal immunoglobulins, as previously reported [20, 22]. Although Michallet et al. [20] proposed that HLC monitoring may better identify patients achieving ≥ CR, the present study could not confirm this observation because of the small number of patients attaining CR. Notably, 15 patients who achieved VGPR according to IMWG criteria were reclassified as having PR based on HLC response criteria as their dHLC reduction was less than 90.0%. None of these patients had detectable M-protein levels on SPEP, whereas SIFE remained positive. According to HLC measurements, in some cases, both iHLC and uHLC values, as well as HLCr, returned to within normal ranges; however, the calculated dHLC at response evaluation remained below the 90.0% reduction threshold. Because dHLC quantifies the difference between involved and uninvolved immunoglobulin pairs, it may detect subtle residual imbalances that are not apparent when absolute immunoglobulin levels are considered alone. These findings suggest that the HLC assay may provide complementary information to conventional methods for treatment response assessment, particularly in cases with borderline or discordant results, thereby highlighting its potential analytical sensitivity for MM monitoring. Reclassification of treatment response from VGPR to PR may have implications for clinical decision-making and, potentially, for the prediction of patient outcomes. However, the clinical relevance of HLC-based reclassification remains uncertain, and these findings should be interpreted with caution. At present, the observed discordance should be considered hypothesis-generating; prospective studies incorporating clinical outcomes are required to determine whether HLC-guided response assessment provides incremental value beyond established IMWG criteria.

The analytical sensitivity assessment further supported the robustness of the HLC method. Our results demonstrated excellent correlation and strong agreement between HLC and total immunoglobulin measurements, consistent with previous studies validating the analytical performance and reproducibility of HLC assays [17, 22]. Although HLCr showed excellent concordance with SIFE results, both HLCr and iHLC demonstrated poor agreement with the SFLC ratio. This discordance between HLC parameters and the SFLC ratio likely reflects intrinsic biological differences between the assays, as they quantify distinct components of immunoglobulin synthesis and secretion, rather than indicating analytical superiority of one method over another.

When comparing the analytical sensitivity for detecting M-protein among SPEP, SIFE, and HLC assays, SPEP remains the most widely used screening method for monoclonal gammopathies; however, it has the lowest sensitivity, with a detection limit of approximately 0.2–0.5 g/dL, depending on the migration characteristics of the monoclonal immunoglobulin [34–37]. SIFE provides greater sensitivity than SPEP, with a detection limit of approximately 0.1 g/dL. However, as a qualitative or semi-quantitative technique, interpretation of faint monoclonal bands may be subjective, particularly during response monitoring or MRD assessment [5, 34–37]. In the present study, SIFE detected monoclonal bands at concentrations as low as 0.0063 g/dL. In contrast, the HLC assay remained quantifiable at all tested dilutions, with detection limits of 0.0009 g/dL for IgGκ and 0.0005 g/dL for IgGλ, as indicated by the manufacturer [17], representing at least a tenfold increase in analytical sensitivity compared with SIFE.

Overall, the present findings indicate that the HLC assay provides practical advantages for quantitative monitoring of MM, particularly in detecting residual disease and assessing the depth of response. However, complete replacement of conventional SIFE and SFLC assays is not justified, as each method provides complementary information. SIFE remains essential for qualitative confirmation of monoclonality, whereas SFLC retains the highest sensitivity for light chain–only disease. Notably, several cases demonstrated detectable M-proteins by the HLC assay, despite being undetectable by SPEP, underscoring its superior analytical performance in low-concentration settings. Nevertheless, quantification of M-proteins by both SPEP and HLC may be influenced by the presence of polyclonal background, particularly in samples with MRD. Moreover, because the HLC assay is clone-specific, it may fail to detect newly emerging or secondary monoclonal components resulting from clonal evolution, development of independent plasma cell clones, or light chain escape during disease progression [16, 17, 19, 20, 22, 35]. Consequently, reliance solely on HLC measurements could lead to underestimation of disease activity in such cases, underscoring the importance of combining HLC testing with conventional methods, such as SPEP, SIFE, or SFLC, to ensure comprehensive monitoring.

This study has certain limitations that should be considered when interpreting the findings. First, the retrospective design and single-center setting may limit the generalizability of the results. Additionally, HLC analysis was performed in a relatively small and selected subgroup of patients with intact immunoglobulin MM, most of whom had already achieved VGPR. This selection may introduce selection bias and further limit the generalizability of the findings, particularly to patients in earlier phases of treatment. Accordingly, larger prospective studies including more heterogeneous patient populations are required to validate the generalizability of these observations. Second, because the analyses were performed using specific reagents and analyzers, cross-platform comparisons of concordance and discordance rates were not feasible. Third, within the HLC assessment cohort, the number of patients achieving CR was limited, constraining evaluation of the assay’s role in minimal residual disease detection. Finally, longitudinal clinical outcome data, such as relapse or survival correlations, were not available; inclusion of such data would have provided additional insight into the prognostic significance and clinical utility of HLC-based monitoring.

Despite these limitations, this study represents one of the few comprehensive evaluations of concordance and discordance between SIFE and SFLC assays in a large cohort of patients with MM, providing real-world insight into their diagnostic interrelationships. The inclusion of the HLC assay enabled direct analytical comparison with conventional methods, demonstrating its quantitative performance and sensitivity across different response categories. All assays were conducted under standardized laboratory conditions using consistent methodologies to ensure internal validity and reliable cross-assay comparisons. Collectively, these methodological strengths enhance the robustness of the findings and support the role of HLC testing as a complementary tool in routine MM monitoring.

## Conclusions

In conclusion, this large retrospective study provides a comprehensive evaluation of concordance and discordance between SIFE and SFLC assays in MM, offering real-world evidence for their complementary diagnosis and disease monitoring. The findings support integration of HLC testing into existing response assessment strategies to enhance the accuracy of treatment evaluation and facilitate interpretation of discordant results. Further large-scale prospective, multicenter studies are warranted to validate these observations and to establish standardized criteria for incorporating HLC measurements into routine clinical practice for comprehensive MM monitoring.

## Supplementary Information

Supplementary Material 1.

Supplementary Material 2.

Supplementary Material 3.

## Data Availability

The datasets generated during and/or analysed during the current study are available from the corresponding author on reasonable request.
