# Supplementary material for: Discordance between immunofixation and free light chain assays in multiple myeloma: a retrospective analysis and evaluation of the heavy/light chain assay for disease monitoring
Source: Blood Res. 2026 Mar 6;61(1):16. doi: 10.1007/s44313-026-00130-9 (PMC13076831; doi:10.1007/s44313-026-00130-9)
Supplement: Supplementary file 1 — Supplementary Material 1. [file 44313_2026_130_MOESM1_ESM.docx]

**Supplementary Table S1** Clinical characteristics of patients with MM included in this study.

|  |  | **Number of patient, n (%)** | | |
| --- | --- | --- | --- | --- |
| **Characteristics** |  | **Comprehensive diagnostic cohort**  **(n = 215)** | **HLC analytical cohort**  **(n = 38)** |  |
| **Age, year** | Mean (±SD) | 62.4 ± 12.2 | 59.3 ± 12.4 |  |
| **Sex** |  |  |  |  |
|  | Male | 120 (55.8) | 13 (34.2) |  |
|  | Female | 95 (44.2) | 25 (65.8) |  |
| **Clonal isotype** |  |  |  |  |
|  | IgG Kappa | 76 (35.3) | 15 (39.5) |  |
|  | IgG Lambda | 51 (23.7) | 11 (28.9) |  |
|  | IgA Kappa | 47 (21.9) | 8 (21.1) |  |
|  | IgA Lambda | 37 (17.2) | 4 (10.5) |  |
|  | IgM Kappa | 1 (0.5) | 0 (0.0) |  |
|  | IgM Lambda | 1 (0.5) | 0 (0.0) |  |
|  | IgD Lambda | 2 (0.9) | 0 (0.0) |  |
| **ISS-stage** |  |  |  |  |
|  | I | 44 (20.5) | 6 (15.8) |  |
|  | II | 55 (25.6) | 11 (29.0) |  |
|  | III | 116 (53.9) | 21 (55.2) |  |
| **ASCT eligibility** |  |  |  |  |
|  | Eligible (age <65 yr) | 125 (58.1) | 28 (73.7) |  |
|  | Non-eligible (age ≥65 yr) | 90 (41.9) | 10 (26.3) |  |
| **First line treatment regimen** |  |  |  |  |
|  | Bortezomib-based regimen | 149 (69.3) | 30 (78.9) |  |
|  | Other regimen | 51 (23.7) | 8 (21.1) |  |
|  | No treatment/palliative care | 15 (7.0) | 0 (0.0) |  |
| **Median follow-up, month** | Median (range) | 9 (1–50) | 11 (6–22) |  |

ASCT, autologous stem cells transplantation; HLC, heavy/light chain; Ig, immunoglobulin; ISS, the International Staging System; SD, standard deviation
