# Supplementary material for: Discordance between immunofixation and free light chain assays in multiple myeloma: a retrospective analysis and evaluation of the heavy/light chain assay for disease monitoring
Source: Blood Res. 2026 Mar 6;61(1):16. doi: 10.1007/s44313-026-00130-9 (PMC13076831; doi:10.1007/s44313-026-00130-9)
Supplement: Supplementary file 2 — Supplementary Material 2. [file 44313_2026_130_MOESM2_ESM.docx]

**Supplementary Table S2** Distribution of SIFE and SFLC results according to treatment response status in 536 samples obtained from 215 patients with multiple myeloma.

| **Treatment response status** | **Test status, *n* (row%)** | | | | **Total,**  ***n* (%)** |
| --- | --- | --- | --- | --- | --- |
|  | **SIFE+/SFLC+** | **SIFE+/SFLC-** | **SIFE-/SFLC+** | **SIFE-/SFLC-** |  |
| All patients | 301 (56.2) | 100 (18.7) | 14 (2.6) | 121 (22.5) | 536 (100.0) |
| At diagnosis | 97 (94.2) | 6 (5.6) | 0 (0.0) | 0 (0.0) | 103 (19.2) |
| PR | 158 (95.8) | 7 (4.2) | 0 (0.0) | 0 (0.0) | 165 (30.8) |
| VGPR | 43 (33.3) | 86 (66.7) | 0 (0.0) | 0 (0.0) | 129 (24.1) |
| CR | 0 (0.0) | 0 (0.0) | 10 (100.0) | 0 (0.0) | 10 (1.9) |
| sCR | 0 (0.0) | 0 (0.0) | 0 (0.0) | 121 (100.0) | 121 (22.5) |
| Relapsed disease | 3 (37.5) | 1 (12.5) | 4 (50.0) | 0 (0.0) | 8 (1.5) |

CR, complete remission; PR, partial response; sCR, stringent complete remission; SFLC, serum free light chain; SIFE, serum immunofixation electrophoresis; VGPR, very good partial response.
