# Supplementary material for: Discordance between immunofixation and free light chain assays in multiple myeloma: a retrospective analysis and evaluation of the heavy/light chain assay for disease monitoring
Source: Blood Res. 2026 Mar 6;61(1):16. doi: 10.1007/s44313-026-00130-9 (PMC13076831; doi:10.1007/s44313-026-00130-9)

**Supplementary file:** Original SIFE figures


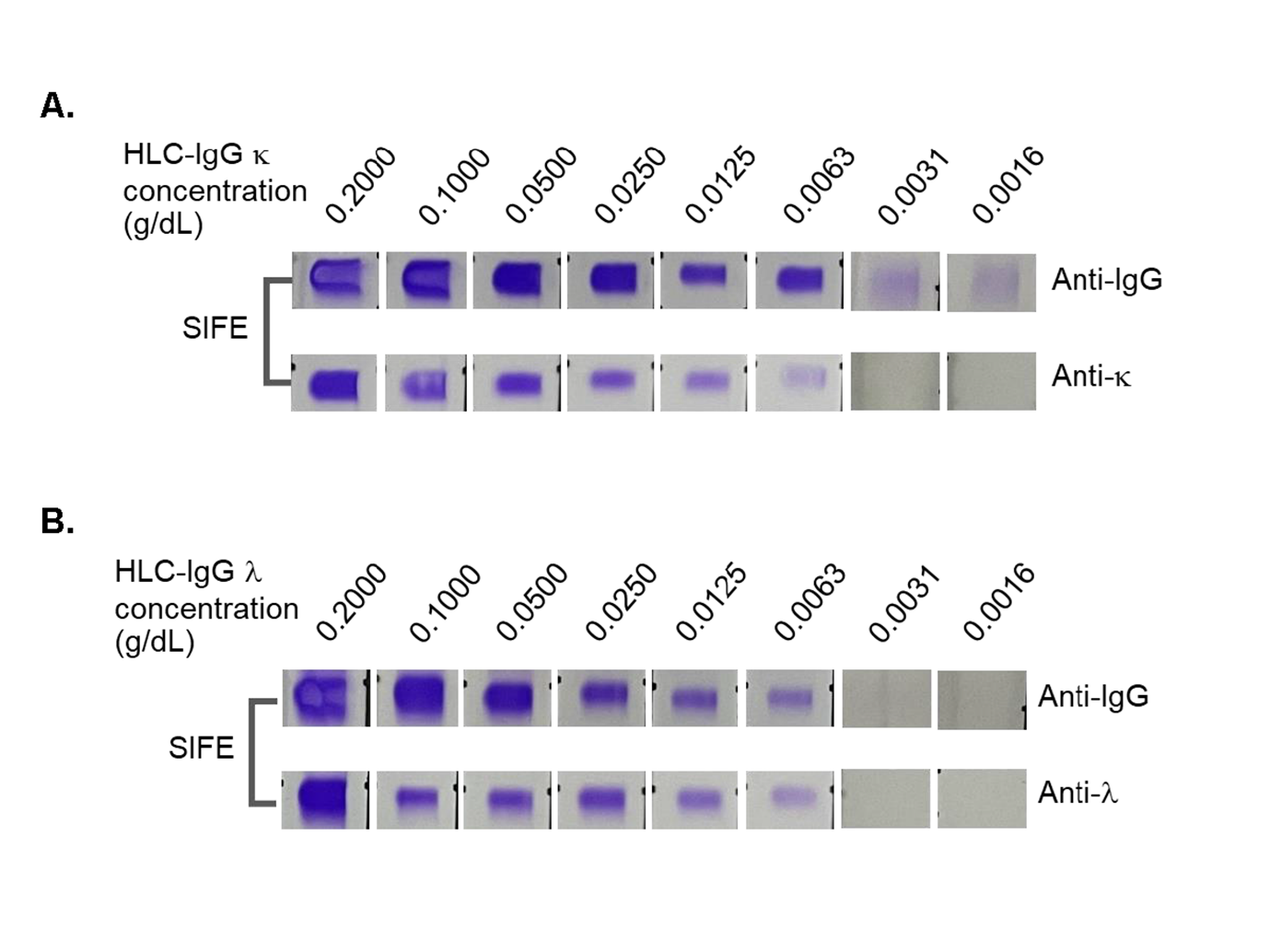


**C.**

**B.**

**Figure 4.** Analytical sensitivity of the HLC assay and SIFE in serially diluted IgGκ and IgGλ samples. Representative serum samples containing monoclonal (A) IgGκ and (B) IgGλ proteins were serially diluted from an initial concentration of 0.200 g/dL. he SIFE bands became faint and undetectable at concentrations below 0.0063 g/dL, whereas the HLC assay remained quantifiable across all tested dilutions, consistent with its lower limits of detection (0.0009 g/dL for IgGκ and 0.0005 g/dL for IgGλ).

**Supplementary file:** Original SIFE figures


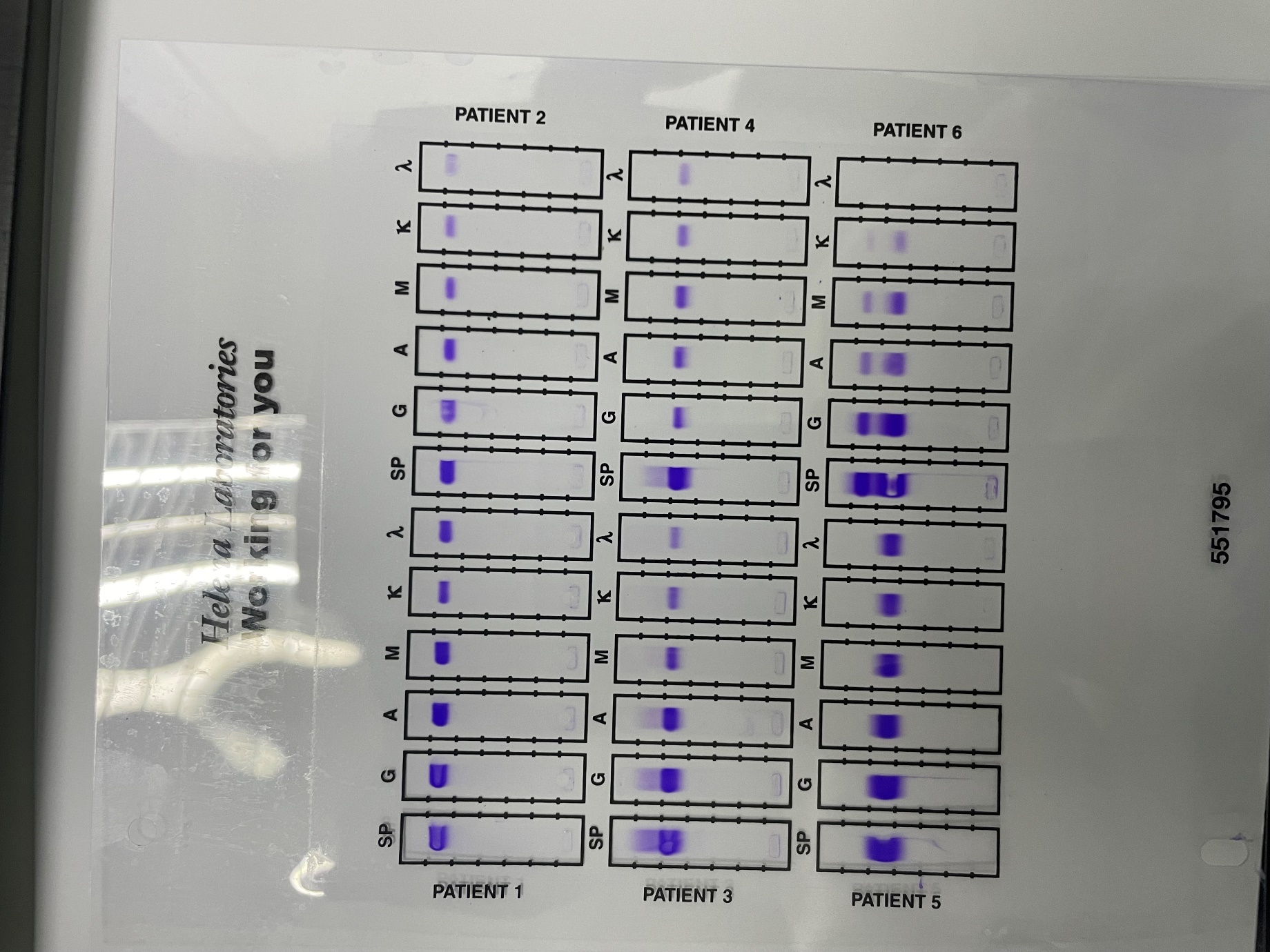


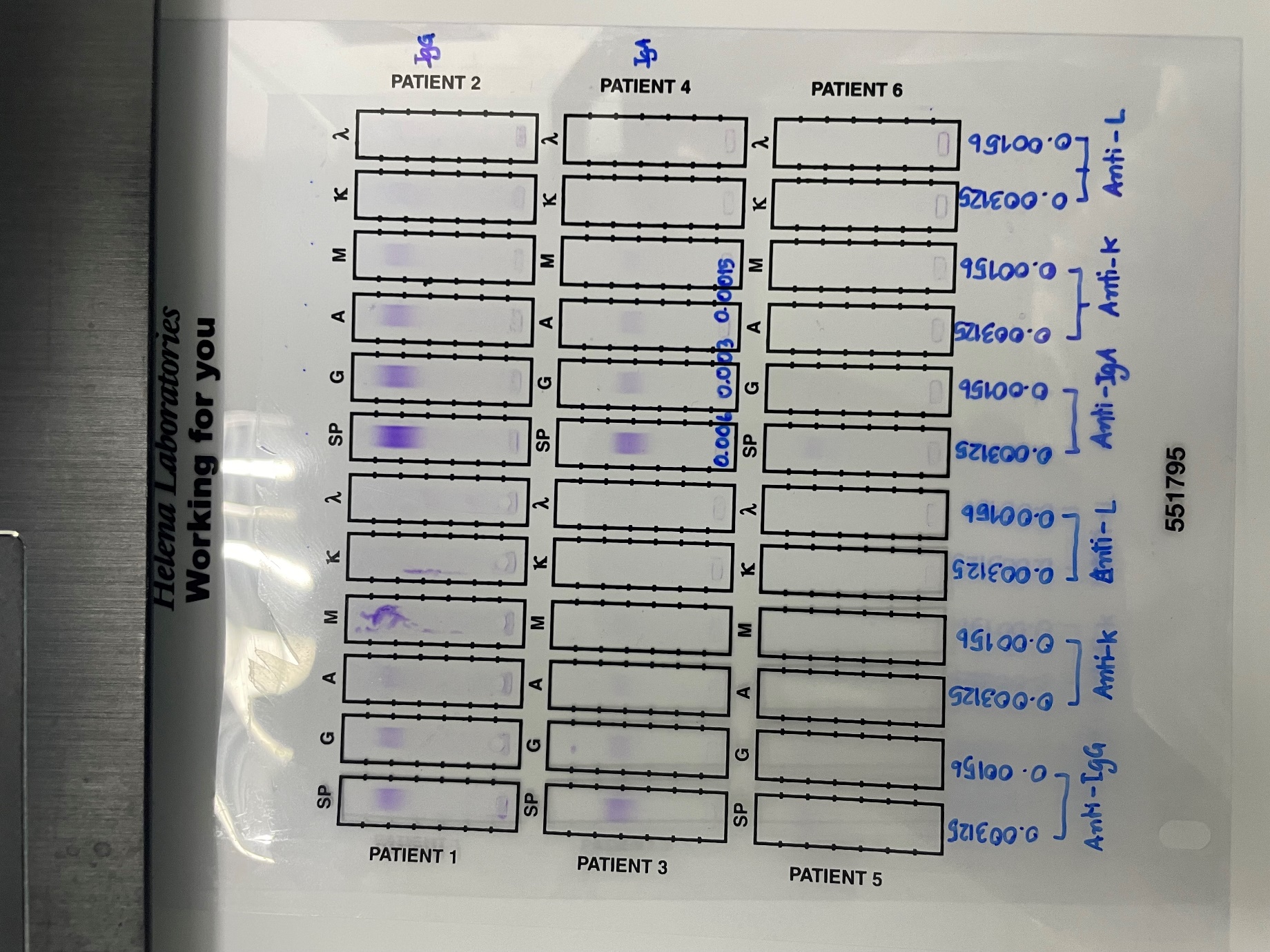

Supplement: Supplementary file 3 — Supplementary Material 3. [file 44313_2026_130_MOESM3_ESM.docx]
